# Prevalence and Associated Factors of Violence against Hospital Staff at Amanuel Mental Specialized Hospital in Addis Ababa, Ethiopia

**DOI:** 10.1155/2019/3642408

**Published:** 2019-11-05

**Authors:** Ayalew Abate, Dessie Abebaw, Addis Birhanu, Aemero Zerihun, Dawit Assefa

**Affiliations:** ^1^Federal Ministry of Health, Amanuel Mental Specialized Hospital Addis Ababa, Ethiopia; ^2^University of Gondar College of Medicine and Health science, Institute of Public Health, Ethiopia

## Abstract

*Background. *Violence at the workplace has become an alarming phenomenon worldwide. The real size of the problem is largely unknown and recent information shows that the current knowledge is only the tip of the iceberg. The enormous cost of violence at the workplace for person and community at large is becoming more apparent. It could be physical, sexual, and verbal in nature and could be actual or threatened. *Objectives. *To access prevalence and associated factors of violence against hospital staff at Amanuel Mental Specialized Hospital Addis Ababa, Ethiopia. *Methods. *An institution based cross-sectional study was employed in 2017. The data were collected using Workplace Violence in the Health Sector Country Case Study Questionnaire from 496 participants. Participants had been selected using simple random sampling technique and data were collected using a self-administered structured questionnaire. The collected data were entered into Epi-data version 3.1, and SPSS version 21 was used for Analysis. Binary logistic regression was fitted to identify factors associated with the outcome variable. *Result. *From 496 staff intended to have participated in this study, complete data were obtained from 435, making a response rate of 87.7%. This research showed high prevalence of violence and we have got that staff had been exposed to physical violence 36.8%, verbal violence 62.1%, and sexual violence 21.8 % over the past year, respectively. Age, sex, and contact with the patient were statistically significant variables (*p* < 0.05). *Conclusion and Recommendation. *According to this study, majority of AMSH staff were violated by the patient they care.

## 1. Introduction

Violence is defined as being destructive towards another person. It is expressed as physical assault, homicide, verbal abuse, bullying, sexual harassment, and mental stress [1–4]. Globally, violence is recognized as a major public health issue and which is a leading cause of death for people aged 15–44 years. Worldwide data showed that millions of people get violated at their work place and this is an emerging problem [5, 6]. Workplace violence affects every individual regardless of their perspective profession. All categories of healthcare workers are at risk of violence. Evidence suggests that the health care environment can be inherently more challenging than other nonhealth sectors and exposed for violence [2–4].

A study conducted in Switzerland showed that aggression and violence are major problem in acute psychiatric wards with career prevalence rates of being assaulted approximating 100% for mental healthcare staff [7]. A study conducted in Italy showed that one out of ten workers report physical assault and one out of three exposed to nonphysical violence in the workplace. Nurses and physicians were the most exposed occupational categories, whereas the psychiatric and emergency departments were the services at greatest risk of violence [8]. In Italy, violent incidents more frequently occurred in psychiatry department (86%), emergency department (71%), and in geriatric wards (57%) [9]. In china verbal violence against nurses were 46% [10]. In North China, physical violence against nurses were 7.8% and 71.9% reported nonphysical violent [11].

A systematic review and meta-analysis conducted in a high income country showed that the pooled proportion of patients who committed at least one act of violence was 17%. According to the review 1 in 5 patients admitted to acute psychiatric units commit an act of violence [12]. A follow up study conducted in public health and mental health units showed that the relationship between work-related distress and work place violence is bidirectional. Having bad health outcome at follow-up, nonphysical violence, a prolonged state of stress, and social isolation were significant predictors of workplace violence [12–14].

Study findings in Iran revealed that 44.3% patients were the source of physical abuse and for 55.6% the members of patients' family were the source. In 30.3% cases the patients were the source of verbal violence and in 53.4% cases the members of patients' family were the source and in 16.1% cases coworkers were the sources [15]. The study conducted in 2014 at Nigeria showed that the highest prevalence was among the nurses 53.5% and the commonest forms of assault were verbal 64.6% and physical abuse 35.4% the violent acts mostly occurred at the accidents and emergency 30.6% [2, 6].

Study conducted in Lebanon during 2015 revealed that: prevalence of nurses exposed to verbal abuse was 62% and physical violence was 10%. Among respondents, 31.7% of nurses indicated likelihood to quit their jobs and 22.3% were undetermined [16, 17]. A cross sectional study conducted in Palestinian hospital showed that 80.4% respondents reported exposure to violence; of which were 20.8% physical and 59.6% nonphysical. Variables like sex, level of education, and workplace were predictor variables for violence [18]. In Malawi 86% of nurses were violated; of this verbal abuse (95%), threatening behaviors (73%), physical assaults (22%), sexual harassment (16%), and others (3%). Perpetrators of violence were: patients (71%), patients' relatives (47%), and work colleagues (43%) [19]. Study conducted in Jordan revealed that verbal violence account for 95.3% and 23.3% physical violence [11]. Study conducted among Iranian healthcare professionals who worked in teaching hospitals in 2011 reported as about 75% of the participants had been subjected to workplace psychological violence (76.1%) [20, 21].

In Sub-Saharan Africa, researchers reported that prevalence of violence in the health care sector was very high up to 88%. In health sectors bullying and harassment were more prevalent and common [2]. Factors like aggression, poor interpersonal interactions, shift work, dysfunctional team, poor communication, low level of support, feeling of being poorly managed, and conflicts can increase vulnerability for violence particularly in psychiatric hospital [1, 2].

**Figure 1:**
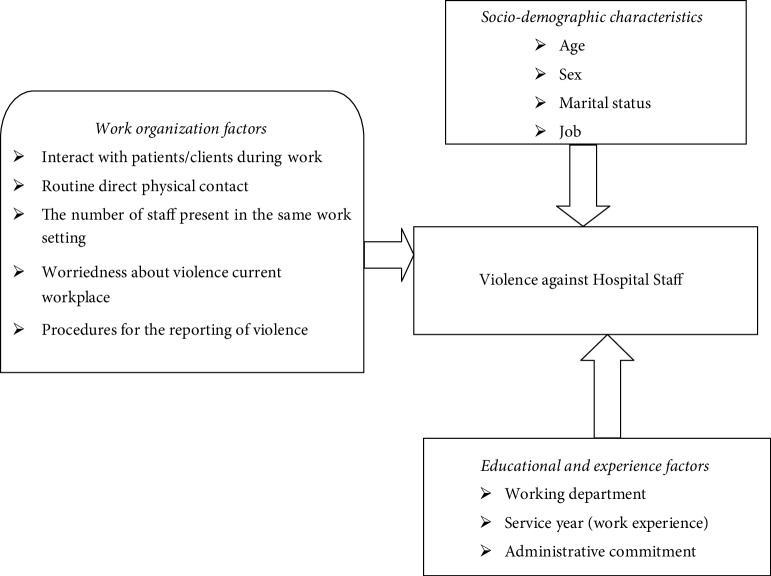
Conceptual Framework.

The effects of violence can be minor physical injuries, serious physical injuries, temporary and permanent physical disability, psychological trauma, and death. Workplace Violence leads to have low worker morale, job stress, worker turnover, reduced trust of management and colleagues, and hostile working environment [22] (Figure 1).

Recent data indicate that hospital workers are at high risk of experiencing violence in the workplace. Several studies indicate that violence often takes place during times of high activity and interaction with patients, such as at meal times and during visiting hours and patient transportation. Assaults may occur when service is denied, when a patient is involuntarily admitted, when a health care worker attempts to set limits on eating, drinking, tobacco, or alcohol use [22]. Workplace Violence in acute psychiatric wards affects the safety of other patients, staff, and the effectiveness of treatment [12].

Violence at work has become an alarming phenomenon in Ethiopia. Cost of violence at work place is becoming more apparent for individual and community at large. This violence behavior is more violent and common in Amanuel Mental Specialized Hospital. Therefore, this study was planned to assess the magnitude of violence and factor associated among staff.

### 1.1. Justification of the Study

In developing country including Ethiopia violence against hospital staffs by mentally ill patients is not well-known despite the high prevalence and burden of violence. Even though there are few studies in different parts of the world like Europe, Asia and America, no study done in Ethiopia.

Since it is the first research done on psychiatry hospital staff in Ethiopia, the finding of this study can serve as a baseline to provide government bodies, nongovernmental organizations, policy makers and health planners with relevant information for future planning and interventions of appropriate strategies to prevent the consequences of violence.

## 2. General Objective

To assess prevalence of violence against Hospital staff at Amanuel Mental Specialized Hospital in Addis Ababa, during a period of a month (November 2017).

### 2.1. Specific Objective

To identify associated factors of violence against hospital staff at Amanuel Mental Specialized Hospital.

## 3. Study Design and Period

This institutional based cross-sectional study was conducted at Amanuel Mental Specialized Hospital Addis Ababa, from 1 to 30 November 2017. This hospital is the only specialized hospital in Ethiopia that gives service for more than 100 million populations.

### 3.1. Inclusion and Exclusion Criteria

All AMSH permanently working staff were included in the study. Those who were not at work and with less than one service year were excluded from the study.

### 3.2. Sample Size Determination

The adequate number of samples required for this study was determined by using Single population proportion formula considering the following assumptions. The level of significance was taken as 95%, (*Zα*_2_ = 1.96), margin of error 0.3%. In addition to compensate for nonresponses rate, 10% were added. The formula used was as the following:

(1) $$\begin{array}{c} n=\frac{{\left(Z\alpha /2\right)}^{2}\cdot P\left(1-P\right)}{d^{2}}, \end{array}$$

where, *P* = expected proportion of violence towards health workers 88% [2], *Zα*/2 = critical value at 95% confidence level of certainty (1.96). *d* = the margin of error between the sample and population. *n* is the required sample size so the sample size were

(2) $$\begin{array}{c} n=\frac{{\left(1.96\right)}^{2}\cdot 0.88\left(1-0.88\right)}{{\left(0.03\right)}^{2}}=451. \end{array}$$

Therefore *n* = (451) since N < 10,000, using single population correction formula, Assuming nonresponse rate (NR), incomplete questionnaires, and lost filed questionnaires around 10%. 451 × 10% = 45.1, Therefore, the total sample size =451 + 45.1 = *n* = 496.

### 3.3. Sampling Technique

Simple random sampling method was used to get the required sample size. Strata created based on profession and sample within each stratum was further selected by simple random sampling. There were 2 categories of the staff in hospital, which was health profession and nonhealth profession and fulfilled the inclusive criteria and stratified.

### 3.4. Dependent Variable

Violence against hospital staff.

### 3.5. Independent Variables

Sex, Age, Marital status, Service year, job (Profession), Personnel interaction, interact with patients/clients during work, routine direct physical contact, number of staff present in the same work setting, worriedness about violence current workplace, procedures for the reporting of violence.

### 3.6. Data Collection Procedure

Self-administered questionnaire was used. The instrument comprised of socio-demographic data; ILO/ICN/WHO/PSI Workplace Violence in the Health Sector Country Case Study—Questionnaire which assesses violence and ill treatment from patients, perceived causes of violence/assault, their response/reactions to violence and the hospital procedure of handling such and also the respondent suggestions to address the threat for which the reliability was confirm through different literatures including African countries [2, 3, 5].

### 3.7. Data Collectors' Technique

The data collectors had been given a general introduction to the study as well as the opportunity to ask questions about the study and complete Workplace Violence in the Health Sector Country Case Study—Questionnaire. A measure of violence against hospital staff: Ten staffs were distributed questionnaires at the working office for those who gave consent after clear explanation of the objectives of the study and confidentiality. The principal investigator and the supervisor checked the completed questionnaires for consistency and completeness on a daily basis.

### 3.8. Data Quality Assurance

A one-day training of data collectors was given on how to collect data. The data collection methods, tools, and how to handle ethical issues had been discussed with the data collectors. Pretest was conducted on 5% of the sample size before the main study to identify potential problems in the proposed study such as data collection tools and to check the performance of the data collectors. Questionnaires used in the pre-test were not included in the analysis as part of the main study. English version questionnaire was used for data collection. Regular supervision by the supervisor and principal investigator was made to ensure that all necessary data were properly collected.

### 3.9. Data Processing and Analysis

Once all necessary data were obtained, data were checked for completeness and a particular questionnaire with incomplete data had been assessed. Data were edited, cleaned, coded, and entered into, and analyzed by SPSS version 21 for windows. Binary Logistic regression was fitted with the data. Then Bivariate and multivariable logistic regressions had been used to identify the independent predictors of violence. Variables that showed statistically significant association with *p*-value of less than 0.25 on bivariate analysis had been entered into multivariate logistic regression. The variables which have a statistical significance association were identified on the basis of *p*-values <0.05 and AOR with 95% confidence intervals. The model fitness for multivariable logistic regression was checked by Hosmer and Lemeshow for goodness-of-fit test and maximum likelihood ratio or Chi-square difference test.

### 3.10. Operational Definitions

#### 3.10.1. Workplace Violence

Workplace violence ranges from offensive or threatening language to homicide. As violent acts (including physical assaults and threats of assaults) directed towards persons at work or on duty.

#### 3.10.2. Physical Violence

The use of physical force against another person or group that results in physical, sexual, or psychological harm; includes beating kicking, slapping, stabbing, shooting, pushing, biting, and pinching among others.

#### 3.10.3. Psychological Violence

Intentional use of power, including threat of physical force, against another person or group, that can result in harm to physical, mental, spiritual, moral, or social development; Includes verbal abuse, bullying/mobbing, harassment, and threats.

#### 3.10.4. Threats

Expressions of intent to cause harm, including verbal threats, threatening body language, and written threats.

#### 3.10.5. Physical Assaults

Attacks ranging from slapping and beating to rape, homicide, and the use of weapons such as firearms, bombs, or knives.

### 3.11. Ethical Consideration

Ethical approval was obtained from Amanuel Mental Specialized Hospital Institutional review Board (IRB). The purpose and importance of the study was explained for study participants and informed the right to withdraw at any time during the study period. Participants were recruited after signing the written informed consent. No personal identification such as name was collected to maintain the privacy and confidentiality of participants.

## 4. Result

### 4.1. Socio Demographic Characteristics

In this study 496 participant were included with response rate of 435 (87.7%). Of the total respondents, 206 (47.4%) were males and 229 (52.6%) were females. The minimum and maximum ages of the respondents were 20 and 58 years respectively with mean and standard deviation of 34.45 ± 11.02 years. According to marital status, 35.4% were single, and 49.9% were married. Concerning the field of work, 71 (16.30%) of the participants were nurse by occupation, 76 (17.47%) of the participants were cleaner. When we saw service year of the staff 115 (26.4%) worked 6–10 years, and 85 (19.5%) were 11–15 years in the hospital while 40 (9.2%) worked more than 20 years. The staff reported that more than 440 (55.2%) of them had interacted with patients/clients during their work and 188 (43.2%) had direct physical contacts (washing, turning, physical examination), while the rest had indirect contact. Based on direct interaction and contact with patients, 240 (55.2%), and 188 (43.2%) had interaction and physical contact, respectively (Table 1).

#### 4.1.1. Violence

The prevalence of workplace violence is presented in Table 2. One hundred sixty (36.8%) reported exposure to physical violence, nearly 270 (62.1%) reported verbal abuse while 95 (21.8%) reported to have been sexually harassed (Table 2).

### 4.2. Physical Violence

From 435 participants 160 (36.78%) reported that they have physical attack by patients/clients, while the rest 275 (63.22%) did not report physical attack. When they received physical attack, they responded differently. The majority of them 38 (23.8%) reported that they tried to defend themselves physically 41 (9.4%), did not take any action, 26 (6.0%) tried to pretend it never happened, 23 (5.3%) informed to stop his/her action, 38 (8.7%) tried to defend themselves, 22 (5.1%) informed it to their friends/families, 22 (5.1%) were given counselling to stop their action,31 (7.1%) told to their colleagues (co-workers), and 10 (2.3%) reported it to senior staff members. In the last 12 month they had witnessed 255 (58.6%) physical attacks in the hospital and 157 (61.6%) experienced once, 91 (35.7%) sometimes, and the least 7 (2.7%) reported as it is experienced all the time in the hospital (Table 3).

#### 4.2.1. Verbal Violence

From 435 participants 270 (62.1%) had verbal abuse and 102 (37.78%) reported that they have been always abused, while 85, 83 (31.5%, 30.7%) some times and once within the last one year, respectively. They had different responses like 107 (39.6%), 59 (21.85%), 72 (26.6%), and 82 (30.37%) did not take any action, tried to pretend it never happened, sought counseling, and told a colleague, respectively reported. Forty nine (18.14%) tried to take an action to investigate the cause of the verbal abuse and 31 (63.26%), 6 (12.24%), and 7 (14.28%) were given verbal warning, prosecuted, and reported to police respectively. The rest 221 (81.85) did not report the reason, not important 81 (36.6%), felt-ashamed 5 (2.26%), felt guilty 12 (5.43%), negative consequence 29 (12.12%), and useless 94 (42.53%). Two hundred sixteen (49.7%) of the participants witnessed psychological problems in the last twelve months and 99 (45.8%) reported 2–4 times, 61 (28.24%) once, and the rest 56 (25.9%) witnessed 5–10 times in the last twelve months (Table 4).

### 4.3. Sexual Harassment

Ninety five % of the participant had sexual harassment in their work setting by patients 65 (70.5%), by colleagues 21 (22.1%), and 44 (46.3%) once, 2–10 times 37 (38.9%), and 14 (14.7%) had more than ten times, 76 (80%) were female. When we see the effect of sexual harassment, 68 (71.6%) extremely reported that “feeling like everything” an effort to do any activities, avoiding thinking about or talking about the attack or avoiding having feelings related to it was 56 (58.9%), repeated, disturbing memories, thoughts, or images of the events was 44 (46%.3%) and being “super-alert” or watchful and on guard was 40 (42.1%) (Table 5).

### 4.4. Factors Associated to Violence

#### 4.4.1. Physical Violence

During Bivariate Binary logistic regression analysis; age, supportive staffs like food maker, secretary and patient care giver, service year, and interaction with patients and staff number in the same working setting were associated factors with physical violence at *p*-value <0.05 (Table 6).

#### 4.4.2. Verbal Violence

During Bivariate Binary logistic regression analysis, age, supportive staff, and interaction with patient and service year were associated factors with verbal violence at *p*-value <0.05 (Table 6).

#### 4.4.3. Sexual Harassment

During Bivariate Binary logistic regression analysis, sex, age, supportive staff, and service year, and interaction and contact with patients were associated sexual harassment significant at *p*-value <0.05 (Table 6).

#### 4.4.4. Independent Predictors of Violence towards AMSH Staff

All variables with *p*-value <0.25 in the bivariate binary logistic regression were entered into multivariate logistic regressions to control confounding variables. Moreover, variables with *p*-value lower than 0.05 remained in the final model and taken as statistically significant as shown in Table 7.

After adjusting for potential confounders using Multivariate binary logistic regression analysis of (physical violence) in which enter method was employed, it was found that, age group between 31 and 35 showed 4 times odds of receiving physical violence compared with age group of 26–30 years (AOR 4.09, 95% CI = 1.54, 10.86), age between 46 and 50 years was 3 times odds physical violence compared with age group of 26–30 (AOR 3.313, 95% CI = 1.2, 9.138) and age greater than 55> (AOR 7.513, 95% CI = 5.882, 9.138) were significantly associated. Pharmacy had 86% protective than working as a nurse. Interaction with patients with working setting at 5 times odds of physical violence compared with those no interaction with patients (AOR 5.017, 95% CI = 2.738−9.190) were significant. The number of staff with 1–5 in the same working area was 64.6% less likely being physically abused compared to a single worker (AOR 0.364, 95% CI = 0.17, 0.778). There was no association between sex, marital status, profession, and contact with patients for physical violence.

For verbal abuse after adjusting for potential confounders using binary logistic regression analysis of (physical violence) pharmacy (AOR 0.123, 95% CI = 0.03, 0.508), interaction with patients at 1.85 times odds of verbal abuse compared with those with no interaction with patients (AOR 1.854, 95% CI = 1.084, 3.173) and staff number in the same work setting between 1 and 5(AOR 0.419, 95% = 0.206, 0.852) were significant.

For sexual abuse (harassment) after adjusting for potential confounders using logistic regression analysis, it was found that age between 20 and 25 years were 2 times odds of victim for sexual abuse (AOR 2.01, 95% CI = 1.43, 2.28). Similarly, the likelihood of sexually abused staff who served 1–5 years were 5.4 times compared with service less than one year (AOR 5.45, 95% CI = 1.894, 15.679). Office workers had 69% protection than working as a nurse for sexual harassments. Interaction with patients at 2.418 times odds of sexual abuse compared with no interaction (AOR 2.418, 95% CI = 1.309, 4.467) and contact with patients at 2.6 times odds during work time compared with no contact with patients (AOR 2.639, 95% CI = 1.502, 4.636) were significant with sexual harassment. But there was no association with sex, marital status, and profession with sexual violence.

## 5. Discussion

This result indicated that the staff had been exposed to physical violence (36.8%), verbal violence (62.1%), and sexual harassment (21.8%), respectively. The magnitude of verbal violence was in line with findings from a study conducted in Gambia (verbal violence 59.8%) and the magnitude of physical violence (17%), and sexual harassment (10%) were higher in our study as compared with the study conducted in Gambia [4]. In addition verbal and physical violence were higher in this study as compared to a study conducted in China with verbal violence of 46% and physical violence of 7.8%. The higher prevalence of physical violence in our study could be explained by personal, societal and institutional factors as well include all staff working in the hospital. The commonest form of assault were verbal which was similar to the study conducted in 2014 at Nigeria showing that the highest prevalence was among the nurses with 53.5% and the commonest forms of assault were verbal 64.6% and physical abuse 35.4% the violent acts mostly occurred at the accidents and emergency 30.6% [2, 5, 6]. The current study finding is lower than that of a study conducted in Switzerland as the prevalence of being assaulted is around 100% and showed that violence is a major problem in acute psychiatric wards with career for mental healthcare staff [7], whereas our finding is higher than a systematic review and meta-analysis conducted in high income country (17%) [12]. Verbal abuse ranges from 57% to 100% according to different literatures. So this is the most common type of violence at workplace [8, 12, 13].

### 5.1. Associated Factors with Violence

#### 5.1.1. Having One or Less Co-Workers

This study finds out that one or less co-workers have the highest risk of reporting verbal abuse and 5.5 times more likely to have sexual harassment than those with 5 or more co-workers. This is supported by similar studies conducted [2–4]. This might be that perpetrators were less inclined to direct verbal aggressions towards staffs with more than 5 members than a ward which has less staff number. The other possible explanation for this is that when the number of staff is low in a given working area, patient care could be delayed resulting in irritation from patients or their accompanying persons.

Our results and findings are different or contradict with an Egyptian study which indicates that about one-third of staff reported exposure to violence even in the presence of more than 10 other colleagues [3]. It might be because of cultural differences and staff collaboration at ward. In our case, staff more collaborated with each other and one protects the other if there are more numbers of staff. 

#### 5.1.2. Being Male

Being male were 53% less likely to have physical violence as compared with being females. This finding is similar to other findings [12, 22]. It might be because of the fact that males have more skill, power and experience to manage this kind of violence than females. In addition to this, client might be frightened of males than females to apply violence over males than females [1, 2]. 

#### 5.1.3. Age

The odd of exposing for physical violence were 4 times higher among staff with age category of 31–35 years, 3.3 times higher among 46–50 age categories, and 7.5 times higher among age group greater than 55 years as compared with age category 26–30 years. This is also supported by other research findings in developed and developing world's [1, 2, 12, 13, 23]. As age gets older and older the risk might be increasing; because people who are aged may not have enough energy and power to manage the violence as compared with young or less aged people [2, 6]. 

#### 5.1.4. Staff Department

Being pharmacy hospital staff was 84% less likely to have physical violence, 88% less likely to have verbal violence as compared with nurse staff. This is supported by other studies conducted in a developed country's Psychiatric wards [2, 6, 23]. The possible explanation might be because of the fact that pharmacy staff are not more exposed and have no direct and routine contact with patients or clients at ward, whereas nurses have more frequent contact and communication and also nurses are responsible to manage the patients at ward. This frequent contact may lead nurses to be violated by patients at ward. 

#### 5.1.5. Personnel Interaction

Staff who have not good personal interaction with clients were 2.6 times higher for having physical violence, 1.38 times higher to have verbal violence, and 2.4 times higher to have sexual violence as compared to with staff who have good personal interaction. This is in line with studies conducted in sub-Saharan Africa [16, 17]. It is because of the fact that having good personal interaction might create good patients and care giver atmosphere and also may create like family relationship, whereas having sexual harassment was 2.6 times higher among staff who have direct contact with clients than those who don't have direct contact. This is also in line with other findings in developing and developed countries [20, 21]. It might be that when staff are closer with clients they might be subjected to sexual harassment. 

#### 5.1.6. Service Year

Having physical violence was 2.6 times higher among staff who have 1–5 service years and having sexual harassment was 5.5 times higher among staff who have 1–5 service years as compared with staff who have less than one service year. This finding is in line with different existing literatures both in developed and developing countries [1–4]. The possible explanation might be; those staff who have more stay at psychiatric hospital might be more exposed to violence, because working a long time with such wards increases the possibility of having physical violence, verbal violence, sexual violence than staff who have less than one-year experience. Even though staff who have a long time experience have more advanced skill to manage and protect him/her from violence, they are at a higher risk of having violence.

### 5.2. Limitations of the Study

Recall bias and social desirability biases were the limitation of the study. The other limitation was Variables like aggressive behavior, expressed emotion, and stress were not included in this study. The clinical and demographic variables of people with psychiatric disorders were not included in this study.

## 6. Conclusion

This study highlighted that violence against staff was a very serious problem in our Psychiatric Hospital, where physical, verbal violence, and sexual harassment committed by patients against staff were very frequent. Hospital staff variables such as patient interaction modality, one or more years of service and age >31 years were statistically significantly correlated with violence. Staff tried to manage violence by counseling the client, communicating with care giver, and informing to stop the action. Therefore, the techniques used by professionals to manage patients who behave with aggressiveness were different but partially adequate to prevent workplace violence.

### 6.1. Recommendation

#### 6.1.1. For Amanuel Mental Specialized Hospital and Ministry of Health

(i) Should assign trained persons how to take measures at emergency, regular OPD and at each inpatient services to protect the staffs as a whole in the compound.
(ii) Need to be assigned Trained Guards specially for nurses and prescribers
(iii) Give training for staffs to have good personal interaction with clients

#### 6.1.2. For Researcher

Future research is recommended to know impact of societal factors on violence in the health sector. As part of such an expanded research agenda, the impact of economic globalization, restructuring, and downsizing on levels of violence, as well as on the general standard of health care provision, should be considered.

## Figures and Tables

**Table 1:** Characteristics of staff participants (*n* = 435).

Item	Job description	Frequency (*n*)	Percent (%)
Sex	Male	206	47.3
	Female	229	52.3
Age	20–25	83	19.1
	26–30	142	32.6
	31–35	36	8.3
	36–40	65	14.9
	41–45	30	6.9
	46–50	37	8.5
	51–55	23	5.3
	>55	19	4.4
Marital status	Single	154	35.4
	Married	217	49.9
	Separated, widowed/divorced	64	14.7
Service year	<1	76	17.5
	1–5	46	10.6
	6–10	115	26.4
	11–15	85	19.5
	16–20	73	16.8
	>20	40	9.2
Health professional	Nurse	70	39.3
	Prescribes	49	27.5
	Pharmacy	22	12.4
	Laboratory	11	6.2
	Psychosocial worker	13	7.3
	Other professionals^∗∗^	13	7.3
Supportive staff	Office workers	94	36.6
	General service	163	63.4
No of staff in the same room	None	31	7.1
	1–5	66	15.2
	6–10	281	64.6
	11–15	9	2.1
	>15	48	11.0

^∗^Separated, widowed/divorced. ^∗∗^Environmental, health officer, anesthesia, health assistants.

**Table 2:** Prevalence of violence among AMSH staff (*N* = 435).

Exposure to violence	Physical violence		Verbal violence		Sexual violence	
	*N*	%	*N*	%	*N*	%
Yes	160	36.8	270	62.1	95	21.8
No	275	63.2	165	37.9	340	78.2

**Table 3:** Effect of physical violence among AMSH staff (*n* = 435).

Item description	Not at all		A little bit		Moderately		Quite a bit		Extremely	
	*N*	%	*N*	%	*N*	%	*N*	%	*N*	%
Repeated, disturbing memories, thoughts, or images of the events?	20	12.5	20	12.5	30	18.8	30	18.8	60	37.5
Avoiding thinking about or talking about the attack or avoiding having feelings related to it?	47	29.4	23	14.4	22	13.8	36	22.5	32	20.0
Being “super-alert” or watchful and on guard?	38	23.8	48	30.0	32	20.0	20	12.5	22	13.8
Feeling like everything you did was an effort?	50	31.3	11	6.9	19	11.9	36	22.5	44	27.5

**Table 4:** Effect of verbal violence on staff participants (*n* = 435).

Item description	Not at all		A little bit		Moderately		Quite A bit		Extremely	
	*N*	%	*N*	%	*N*	%	*N*	%	*N*	%
Repeated, disturbing memories, thoughts, or images of the events?	94	34.8	68	25.2	25	9.25	53	19.6	30	11.1
Avoiding thinking about or talking about the attack or avoiding having feelings related to it?	174	64.4	35	12.9	14	5.2	41	15.2	6	2.2
Being “super-alert” or watchful and on guard?	207	76.6	33	12.2	30	11.1				
Feeling like everything you did was an effort?	169	62.6	34	12.6	10	3.7	34	12.6	23	8.5

**Table 5:** Effect of sexual harassment on staff participants (*n* = 435).

Item description	Not at all		A little bit		Moderately		Quite A bit		Extremely	
	*N*	%	*N*	%	*N*	%	*N*	%	*N*	%
Repeated, disturbing memories, thoughts, or images of the events?	15	15.7	15	15.7	15	15.7	6	6.3	44	46.3
Avoiding thinking about or talking about the attack or avoiding having feelings related to it?	9	9.4	4	4.2	20	21.05	6	6.3	56	58.9
Being “super-alert” or watchful and on guard?	12	12.6	21	22.1	10	10.5	12	12.6	40	42.1
Feeling like everything you did was an effort?	6	6.3	4	4.2	17	17.9	-	-	68	71.6

**Table 6:** Bivariate Binary logistic regression of violence among AMSH staffs (*n* = 435).

Characteristics		Physical violence		Verbal violence		Sexual harassment	
		Yes N (%)	COR (95%–CI)	Yes N (%)	COR (95%-CI)	Yes N (%)	COR (95%–CI)
Sex	Male	106 (44.0)	0.738 (0.505–1.079) [0.117]^∗^	106 (49.3)	1.167 (.801–1.701) [.422]	83 (47.7)	.642 (.428–.962) [.032]^∗^
	Female	135 (56.0)	Reference	109 (50.7)	Reference	91 (52.3)	Reference
Age	20–25 years	38 (15.8)	.869 (.505–1.495) [.611]	42 (19.5)	1.573 (.911–2.717) [.001]^∗^	27 (15.5)	.800 (.461–1.389) [.012]^∗^
	26–30 years	70 (29.0)	Reference	56 (26.0)	Reference	58 (33.3)	Reference
	31–35 years	25 (10.4)	2.338 (1.070–5.108) [.033]^∗^	21 (9.8)	2.150 (1.023–4.521) [.044]^∗^	9 (5.2)	2.492 (1.133–5.482) [.428]
	36–40 years	34 (14.1)	1.128 (.627–2.030) [.688]	29 (13.5)	1.237 (.683–2.240) [.482]	17 (9.8)	1.039 (.572–1.884) [.023]^∗^
	41–45 years	19 (7.9)	1.777 (.789–4.002) [.165]^∗^	11 (5.1)	.889 (.393–2.009) [777]	15 (8.6)	1.556 (.682–3.548) [ .901]
	46–50 years	23 (9.5)	1.690 (.805–3.547) [.165^∗^]	21 (9.8)	2.016 (.969–4.192) [.061]^∗^	16 (9.2)	1.679 (.796–3.539) [.293]
	51–55 years	14 (5.8)	1.600 (.651–3.547) [.306]	20 (9.3)	10.238 (2.906–36.068) [.000]^∗^	20 (11.5)	1.657 (.670–4.098) [.173]
	>55 years	18 (5.5)	8.514 (2.407–14.439) [.005]^∗^	15 (7.0)	5.759 (1.818–18.245) [.003]^∗^	12 (6.9)	19.649 (2.545–151.714) [.274]
Marital status	Married	74 (34.7)	Reference	73 (34.0)	Reference	79 (45.5)	Reference
	Single	128 (53.1)	.643 (.424–.975) [.038]^∗^	108 (50.2)	.910 (.602–1.375) [.653]	70 (40.2)	1.456 (.956–2.217) [.080]
	Others	39 (16.2)	1.085 (.613–1.919).780	34 (15.8)	1.144 (.654–1.999).637	25 (14.4)	1.120 (.631–1.986).699
Hospital staff	Nurse	45 (45.9)	Reference	42 (42.9)	Reference	37 (43.0)	Reference
	Prescribes	25 (25.5)	.579 (.275–1.217) [.149]^∗^	26 (26.5)	.754 (.361–1.575) [.452]	25 (29.1)	.929 (.447–1.929) [0.773]
	Pharmacy	6 (6.1)	.208 (.072–.600) [.004]^∗^	5 (5.1)	.196 (.065–.593) [.004]^∗^	4 (4.7)	.198 (.061–.645) [.007]^∗^
	Laboratory	3 (3.1)	.208 (.051–.857) [.030]^∗^	5 (5.1)	.556 (.155–1.997) [.368]	4 (4.7)	.510 (.137–1.898) [.315]
	Psycho–social worker	9 (9.2)	1.250 (.349–4.474) [.732]	10 (10.2)	2.222 (.561–8.798) [.255]	6 (7.0)	.764 (.233–2.506) [.657]
	Others	10 (10.2)	1.852 (.466–7.359) [.381]	10 (10.2)	2.222 (.561–8.798) [.255]	10 (11.6)	2.973 (.753–11.734) [.320]
	Office work	48 (19.9)	.580 (.307–1.093) [.092]^∗^	39 (18.1)	.473 (.252–.888) [.020]^∗^	21 (12.1)	.257 (.131–.504) [.000]^∗^
	General service	95 (39.4)	.776 (.435–1.386) [.391]	78 (36.3)	.612 (.347–1.080) [.090]^∗^	67 (38.5)	.622 (.354–1.094) [.099]^∗^
Service year	< one year	36 (14.9)	1.444 (.692–3.017) [.328]	33 (15.3)	1.303 (.625–2.717) [.480]	15 (8.6)	3.416 (1.520–7.677) [.414]
	1–5 year	26 (10.8)	1.444 (.807–2.585) [.216]	23 (10.7)	1.002 (.559–1.798) [.994]	21 (12.1)	2.340 (1.185–4.621) [ .003]^∗^
	6–10 years	65 (27.0)	Reference	50 (23.5)	Reference	42 (24.1)	Reference
	11–15 years	49 (20.3)	1.512 (.811–2.819) [.193]^∗^	32 (14.9)	.787 (.419–1.479) [.456]	36 (20.7)	2.988 (1.469–6.077) [.003]^∗^
	16–20 years	39 (16.2)	1.275 (.670–2.426) [.460]	46 (21.4)	2.220 (1.151–4.282) [.017]^∗^	36 (20.7)	3.957 (1.911–8.192) [.000]^∗^
	>20 years	26 (10.8)	2.063 (.936–4.549) [.072]^∗^	31 (14.4)	4.488 (1.881–10.710) [.001]^∗^	24 (13.8)	6.100 (2.612–14.244) [.000]^∗^
Interaction	Yes	162 (67.2)	Reference	134 (62.3)	Reference	127 (73.0)	Reference
	No	79 (32.8)	.328 (.221–.486) [.000]^∗^	81 (37.7)	.328 (.221–.486) [.003]^∗^	47 (27.0)	.283 (.187–.428) [.000]^∗^
Contact	Yes	101 (41.9)	Reference	99 (46.0)	Reference	94 (54.0)	Reference
	No	140 (58.1)	.887 (.606–1.299) [.539]	116 (54.0)	1.256 (.859–1.837) [.239]	80 (46.0)	2.087 (1.412–3.086) [.000]^∗^
Staff number	None	6 (3.1)	.946 (.506–1.768) [.861]	15 (7.0)	.390 (.218–.698) [.002]^∗^	6 (3.4)	.342 (.183–.636) [.001]^∗^
	1–5 staffs	21 (8.7)	.306 (.141–.665) [0.003]^∗^	19 (8.8)	.905 (.431–1.900) [.791]	15 (8.6)	.279 (.111–.700) [.307]^∗^
	6–10 staffs	166 (68.9)	Reference	143 (66.5)	Reference	130 (74.7)	Reference
	11–15 staffs	9 (4.6)	2.730 (.944–7.898) [.064]^∗^	6 (2.8)	1.930 (.473–7.870) [.359]	0	—
	> 15 staffs	29 (12.0)	—	32 (14.9)	1.930 (1.014–3.675) [.045]^∗^	23 (13.2)	1.069 (.579–1.972) [.832]
Worriedness of violence	Not worried	132 (55.2)	Reference	101 (50.0)	Reference	106 (60.9)	Reference
	Sometimes worried	77 (32.0)	.526 (.342–.808) [.003]^∗^	72 (47.1)	.889 (.584–1.354) [.583]	48 (27.6)	.414 (.267–.642) [.600]
	Neutral	7 (2.9)	.279 (.107–.732) [.010]^∗^	7 (3.3)	.538 (.206–1.405) [.206]^∗^	.999	.000 (.000)
	Very worried	24 (10.0)	.346 (.191–.626) [.000]^∗^	35 (16.3)	1.400 (.782–2.507) [.258]	20 (11.5)	.453 (.248–.828) [.310]
Procedures reporting violence	Yes	44 (18.3)	Reference	46 (21.4)	Reference	31 (17.8)	Reference
	No	197 (81.7)	.808 (.504–1.297) [.378]	169 (78.6)	.816 (.509–1.310) [.401]	143 (82.2)	1.232 (.755–2.009) [.404]

**Table 7:** Multivariate logistic regression: Association of socio-demographic factors with violence among staff of AMSH Addis Ababa Ethiopia 2017 (*n* = 435).

Characteristics		Physical violence		Verbal violence		Sexual harassment		
		AOR (95%−CI)	*P*–value	AOR (95%−CI)	*P*–value	AOR (95%−CI)	*P*–value	
Sex	Male	0.472 (0.279–0.798)	0.145	0.938 (0.584–1.505)	0.790	0.646 (0.370–1.130)	0.126	
	Female	Reference		Reference		Reference		
Age	20–25	0.924 (0.439–1.944)	0.834	1.937 (0.953–3.937)	0.068	2.00 (1.438–2.280)	0.001	
	26–30	Reference		Reference		Reference		
	31–35	4.09 (1.54–10.86)	0.005^∗^	2.302 (0.998–5.314)	0.051	0.627 (0.230–1.707)	0.361	
	36–40	1.832 (0.788–4.261)	0.16	0.779 (0.359–1.694)	0.529	0.405 (0.153–1.070)	0.068	
	41–45	1.775 (0.623–5.058)	0.283	0.605 (0.220–1.665)	0.331	3.061 (0.988–9.485)	.053	
	46–50	3.313 (1.201–9.138)	0.021^∗^	1.982 (0.791–4.970)	0.145	3.106 (1.102–8.755)	0.232	
	51–55	1.208 (0.325–4.487)	0.778	5.262 (1.219–22.717)	0.126	24.503 (3.95–152.002)	0.201	
	>55	7.513 (5.882–11.474)	0.001^∗^	5.736 (1.361–24.167)	0.317	2.970 (9.642, 13.736)	0.164	
Marital status	Married	Reference		Reference		Reference		
	Single	0.619 (0.338–1.135)	0.121	0.807 (0.458–1.424)	0.460	2.049 (1.076–3.903)	0.129	
	Others	0.823 (0.382–1.776)	0.620	1.695 (0.814–3.527)	0.158	2.001 (0.81–4.941)	0.133	
Hospital staff	(1) Nurse	Reference		Reference		Reference		
	(2) Prescribes	0.510 (0.202–1.287)	0.154	0.931 (0.377–2.301)	0.877	0.642 (0.263–1.563)	0.329	
	(3) Pharmacy	0.160 (0.040–0.637)	0.019^∗^	0.123 (0.030–0.508)	0.004^∗^	0.137 (0.033–0.574)	0.107	
	(4) Laboratory	0.122 (0.013–1.126)	0.064	0.514 (0.095–2.766)	0.438	0.459 (0.077–2.721)	0.391	
	(5) Psychosocial worker	2.593 (0.621–10.836)	0.192	2.814 (0.607–13.046)	0.186	1.135 (0.287–4.493)	0.857	
	(6) Others	2.547 (0.499–13.010)	0.261	1.216 (0.245–6.034)	0.811	2.208 (0.441, 11.047)	0.335	
	(7) Office work	0.717 (0.303–1.697)	0.45	0.535 (0.241–1.188)	0.124	0.31 (0.128–0.749)	0.009^∗^	
	(8) General service	0.777 (0.32–1.886)	0.577	0.789 (0.355–1.755)	0.562	0.851 (0.351–2.065)	0.722	
Service year	(1) < one year	Reference		Reference		Reference		
	(2) 1–5 year	2.623 (1.045–6.585)	0.04^∗^	1.382 (0.603–3.170)	0.445	5.450 (1.894–15.679)	0.002^∗^	
	(3) 6–10 years	1.076 (0.447–2.591)	0.87	0.943 (0.427–2.081)	0.884	2.474 (.911–6.719)	0.076	
	(4) 11–15 years	0.72 (0.284–1.822)	0.488	0.666 (0.286–1.551)	0.345	2.375 (0.817–6.902)	0.112	
	(5) 16–20 years	0.375 (0.137–1.025)	0.056	2.081 (0.851–5.088)	0.108	3.514 (1.119–11.038)	0.231	
	(6) >20 years	1.677 (0.457–6.153)	0.436	2.953 (0.895–9.739)	0.075	3.235 (0.792–13.222)	0.102	
Interaction	(1) Yes	Reference		Reference		Reference		
	(2) No	5.017 (2.738–9.190)	0.000^∗^	1.854 (1.084–3.173)	0.024	2.418 (1.309–4.467)	0.005^∗^	
Contact	(1) Yes	0.797 (0.464–1.369)	0.412	1.483 (0.897–2.453)	0.125	2.639 (1.502–4.636)	0.001^∗^	
	(2) No	Reference		Reference		Reference		
Staff number	(1) None	Reference		Reference		Reference		
	(2) 1–5 staff	0.364 (0.17–0.778)	0.009^∗^	0.419 (0.206–0.852)	0.016^∗^	0.256 (0.109–0.605)		0.002^∗^
	(3) 6–10 staff	3.392 (1.18–9.745)	0.123	1.030 (0.402–2.638)	0.951	0.254 (0.075–0.864)		0.028^∗^
	(4) 11–15 staff	—	—	2.395 (.512–11.197)	0.267	—		—
	(5) > 15 staff	0.718 (0.332–1.554)	0.4	1.719 (.813–3.639)	0.156	0.303 (0.123, 0.747)		0.009^∗^
Violence worried	Not worried	Reference		Reference		Reference		
	Sometimes worried	0.534 (0.312–0.913)	0.222	0.977 (0.590–1.619)	0.928	0.365 (0.205–0.65)	0.801	
	Neutral	0.089 (0.023–0.351)	0.301	0.419 (0.119–1.474)	0.175	—	—	
	Very worried	0.322 (0.153–0.676)	0.103	1.862 (0.943–3.674)	0.073	0.544 (0.256–1.156)	0.113	
Reporting procedure	Yes	Reference		Reference		Reference		
	No	1.295 (0.698–2.404)	0.413	0.632 (0.352– 1.132)	0.123	0.875 (0.458–1.672)	0.686	

∗indicates the significant level which is *p* value less than 0.05.^∗^ = *p* value ≤0.05.

## Data Availability

The data used to support the findings of this study are available from the corresponding author upon request.
